# Prevalence of complex root canal morphology in the mandibular first and second premolars in Thai population: CBCT analysis

**DOI:** 10.1186/s12903-021-01822-7

**Published:** 2021-09-16

**Authors:** Paramee Thanaruengrong, Sirinya Kulvitit, Mettachit Navachinda, Pornkawee Charoenlarp

**Affiliations:** 1 Department of Operative Dentistry, Faculty of Dentistry, Chulalongkorn University, 34 Henri-Dunant Road, Wangmai, Pathumwan, Bangkok, 10330 Thailand; 2 Department of Radiology, Faculty of Dentistry, Chulalongkorn University, Bangkok, 10330 Thailand

**Keywords:** Mandibular premolar, Bifurcation, Trifurcation, C-shaped root canal, Thai population, CBCT

## Abstract

**Background:**

Mandibular premolars demonstrate high variability in root canal morphology, especially mandibular first premolars. The purposes of this study were to determine the prevalence of root canal configurations of mandibular premolars according to Vertucci classification in a Thai population.

**Methods:**

Total of 1159 CBCT images of Thai patients who received radiographic imaging at the Department of Radiology, Faculty of Dentistry, Chulalongkorn University in 2017–2018 was evaluated. The data were reported using descriptive statistics and the relationship between the prevalence of the root canal complexities and sex was analyzed using the chi-squared test.

**Results:**

The most common root canal configuration was Vertucci type I, with a 63.1% and 98% prevalence in the mandibular first and second premolars, respectively. More than 98% of mandibular premolars had a single root. The prevalence of a bifurcation was 28.5% and 1.5% in the mandibular first and second premolars, respectively. The prevalence of a trifurcation was 3.2% in the mandibular first premolar. A C-shaped root canal was observed at 23.7% and 0.7% in the mandibular first and second premolars, respectively. The level of branching was mostly found at the middle 1/3 of the root. Bilateral appearance of the same root canal configuration was identified in 80.3% and 95.9% in the mandibular first and second premolars, respectively. There was no relationship between sex and the prevalence of a bifurcation, trifurcation, or C-shaped root canal.

**Conclusion:**

Mandibular first premolars have more root canal complexities than mandibular second premolars. Horizontal tube shift x-ray technique, CBCT, dental operating microscope, and knowledge of root canal configurations have an important role in root canal identification in mandibular premolar with suspected complex root canal morphology.

**Supplementary Information:**

The online version contains supplementary material available at 10.1186/s12903-021-01822-7.

## Background

Bacterial infection is the main cause of pulp and periapical tissue disease, therefore the main goal of root canal treatment is to disinfect and prevent reinfection [1]. The inability to locate, disinfect, and fill the root canal completely can cause endodontic failure [1]. Therefore, the knowledge of root canal anatomy and variations along with clinical and radiographic assessment of the tooth is vital to endodontic success.

**Fig. 1:**
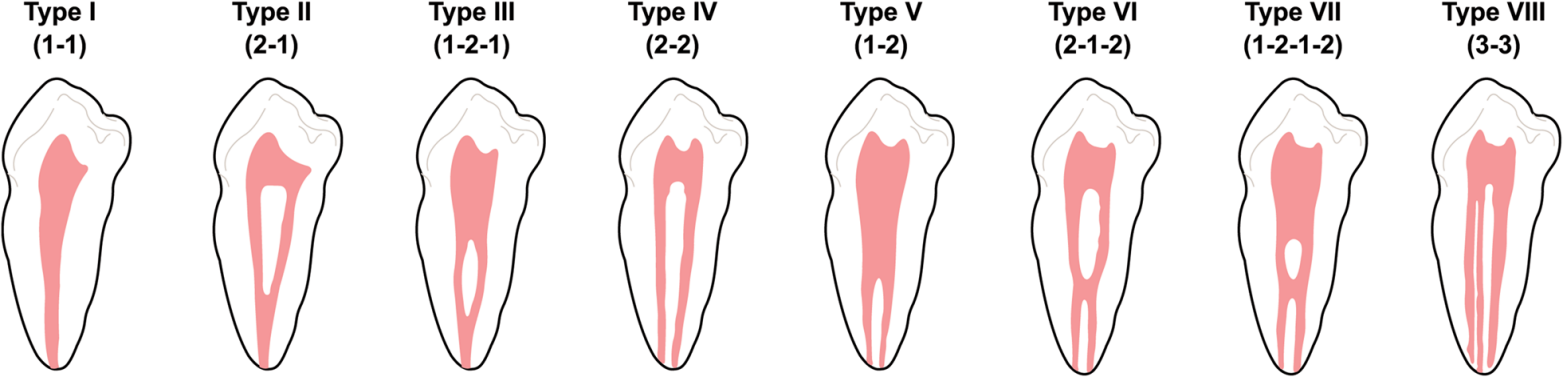
Eight root canal configurations of extracted human permanent teeth classified by Vertucci [2]

Root canal configurations were classified by Vertucci into eight types (Fig. 1) [2]. Type I (1) is a single canal extending from the pulp chamber to the apex. Type II (2-1) is two canals extending from the pulp chamber that join into one canal before reaching the apex. Type III (1-2-1) is one canal that extends from the pulp chamber, divides into two canals, and join into one canal before reaching the apex. Type IV (2) is two canals that extend from the pulp chamber to the apex. Type V (1-2) is one canal extending from the pulp chamber that divides into two canals before reaching the apex. Type VI (2-1-2) is two canals extending from the pulp chamber that join into one canal and then divides into two canals short of the apex. Type VII (1-2-1-2) is one canal that extends from the pulp chamber, divides and then rejoins, and finally divides into two canals short of the apex. Type VIII (3) is three canals that extend from the pulp chamber to the apex.

Ahmed’s classification is a new system for classifying root and root canal morphology [3]. This classification giving detailed information of the root and root canal configuration at the same time. While Vertucci’s classification is more focused on the root canal configuration, which is appropriate for organizing root canal configuration into groups. For example, a tooth with one canal extending from the pulp chamber that divides into two canals before reaching the apex (type V in Vertucci’s classification) can be classified as ^1^TN^1-2^, ^2^TN ^1^B^1^ L^1^, and ^2^TN ^1^M^1^ D^1^ in Ahmed’s classification.

Krasner and Rankow laws are used as a clinical guide for pulp chamber and root canal orifice location. There are two main categories of Krasner and Rankow laws [4]. First, there are 3 laws in “Relationships of the pulp chamber to clinical crown”, which are the law of centrality, law of concentricity, and law of the CEJ. These laws are applied at the CEJ level to aid in pulp chamber floor and walls location. Second, there are 5 laws in “Relationships on the pulp chamber floor”, which are the law of symmetry 1, law of symmetry 2, law of color change, law of orifice location 1, law of orifice location 2, and law of orifice location 3. These laws can be used to identify a root canal orifice.

Mandibular premolars are considered the hardest to treat endodontically due to high variation in their root canal morphology [5, 6]. A University of Washington study assessed the failure rate of nonsurgical root canal treatment in all teeth and found that mandibular first premolars have the highest failure rate at 11.45%. Anatomical variations and difficulties in accessing the lingual canal might explain the high failure rate of mandibular first premolar treatment [7]. Due to the anatomical variations in mandibular premolars, numerous studies have investigated the root canal morphology of these teeth. In a clinical study, root canal morphology was evaluated using two-dimensional periapical radiographs or three-dimensional cone-beam computed tomographic (CBCT) images. However, two-dimensional periapical radiographs are inferior compared with three-dimensional CBCT images in revealing root canal morphology [8–10].

Common anatomical variations in the mandibular premolars are a bifurcation and C-shaped root canal. In contrast, a trifurcation is a rare anatomical variation in the mandibular premolars. A bifurcation is classified as a Vertucci type V root canal configuration, which has a prevalence of 24 and 8% in the mandibular first and second premolar, respectively [5, 6]. In some population, higher incidence of bifurcation was reported in India, Kuwait, Jordan and Turkey [11]. A trifurcation (1–3) is one canal extending from the pulp chamber that divides into three canals before reaching the apex, which has a prevalence 0.4–5% in the mandibular first premolar [12–14] and 0.4% in the mandibular second premolar [14]. A micro-computed tomography study found that mandibular premolars with a trifurcation often have a triangle-shaped pulp chamber [15].

A C-shaped root canal, another common anatomical variation in mandibular first premolars has a prevalence of 1–18% [16–19]. A study also reported that a C-shaped root canal was present in 0.6% of mandibular second premolars [18, 19]. Differences in methodology and variations in sample size and ethnic and regional background of the population may explain the different prevalence between studies, such as the high prevalence of C-shaped root canals found in a Chinese population [18, 20].

Currently there is very limited data on the prevalence and details of root canal morphologies and configurations, including bifurcations, trifurcations, and C-shaped root canals in mandibular premolars, especially in a Thai population [21]. Therefore, the purposes of this study were to determine the prevalence of root canal configurations according to Vertucci classification, number of roots and their location in case of more than 1 root, the prevalence of root canal complexities, including a bifurcation, trifurcation, and C-shaped root canal, level of branching from the main root canal in teeth with multiple root canals, appearance of the radicular groove and its location, bilateral appearance of the same root canal configuration, and the relationship between the prevalence of root canal complexities and sex in a Thai population. This information can guide dentists in the identification and appropriate management of complex root canal anatomy.

## Methods

The study protocol was approved by the Ethics Review Committee on Human Research of the Faculty of Dentistry, Chulalongkorn University (HREC-DCU 2019-053). The CBCT images of mandibular premolars were obtained from CBCT examination performed in Thai patients as part of the diagnosis and treatment plan with a three-dimensional Accuitomo CBCT machine (J Morita Manufacturing Corp, Kyoto, Japan) at the Department of Radiology, Faculty of Dentistry, Chulalongkorn University between January 2017–December 2018.

The sample size was calculated using the n4studies application Version 1.4.1 (Thailand) based on a prior study [19] using the prevalence of C-shaped root canals in mandibular premolars (1.5%) with α value of 0.05 and d (error) value of 0.01. The number of samples required was 568 teeth.

CBCT images of the mandibular first or second premolar that had a field of view of 100 × 100 mm or 100 × 50 mm and had a voxel size of 0.25 mm, were included in the study. The exclusion criteria were the quality of the CBCT image is poor and cannot be analyzed, the information of patients (sex) was missing, and mandibular premolar in the CBCT image had the following properties; previous root canal treatment or having radiopaque material inside the root canal, having a large radiopaque restoration that obscures the visibility of the pulp chamber and root canal, root resorption, incomplete root formation, or a calcified root canal. Furthermore, in case of only one premolar in the quadrant, radiographic anatomy in the coronal view of CBCT images were used to distinguish between first and second premolar as followed; first premolar had a more prominent buccal cusp than underdeveloped lingual cusp, with buccal cusp tip that centered directly over the root, while second premolar had more fully developed lingual cusp [22]. If there were not enough information to identify whether the mandibular premolar in the CBCT image is the mandibular first or second premolar such as inadequate tooth structure, the tooth was excluded from the study.

## Data recording

The CBCT images were displayed on a Dell ST2210 21.5-inch 16:9 aspect ratio flat panel monitor with a screen resolution set at 1920 × 1080 pixels and were evaluated with One volume viewer software (J Morita, Kyoto, Japan) by two independent observers SK, an endodontist with 5 years’ experience and PT, a second-year resident in the endodontic program. If the results did not match, a discussion was held until a conclusion was reached.

SK and PT read the CBCT images of 100 patients and were checked for accuracy by PC, a radiologist, before starting the study. SK and PT read the CBCT images independently. The data recorded were: sex of the patient, tooth number, root canal configuration according to Vertucci classification [2], number of roots and their location in case of more than 1 root, root canal configuration other than Vertucci classification, including a trifurcation and C-shaped root canal; level of branching from the main root canal in the teeth with multiple root canals, appearance of the radicular groove and its location, and bilateral appearance of the same root canal configuration.

**Fig. 2:**
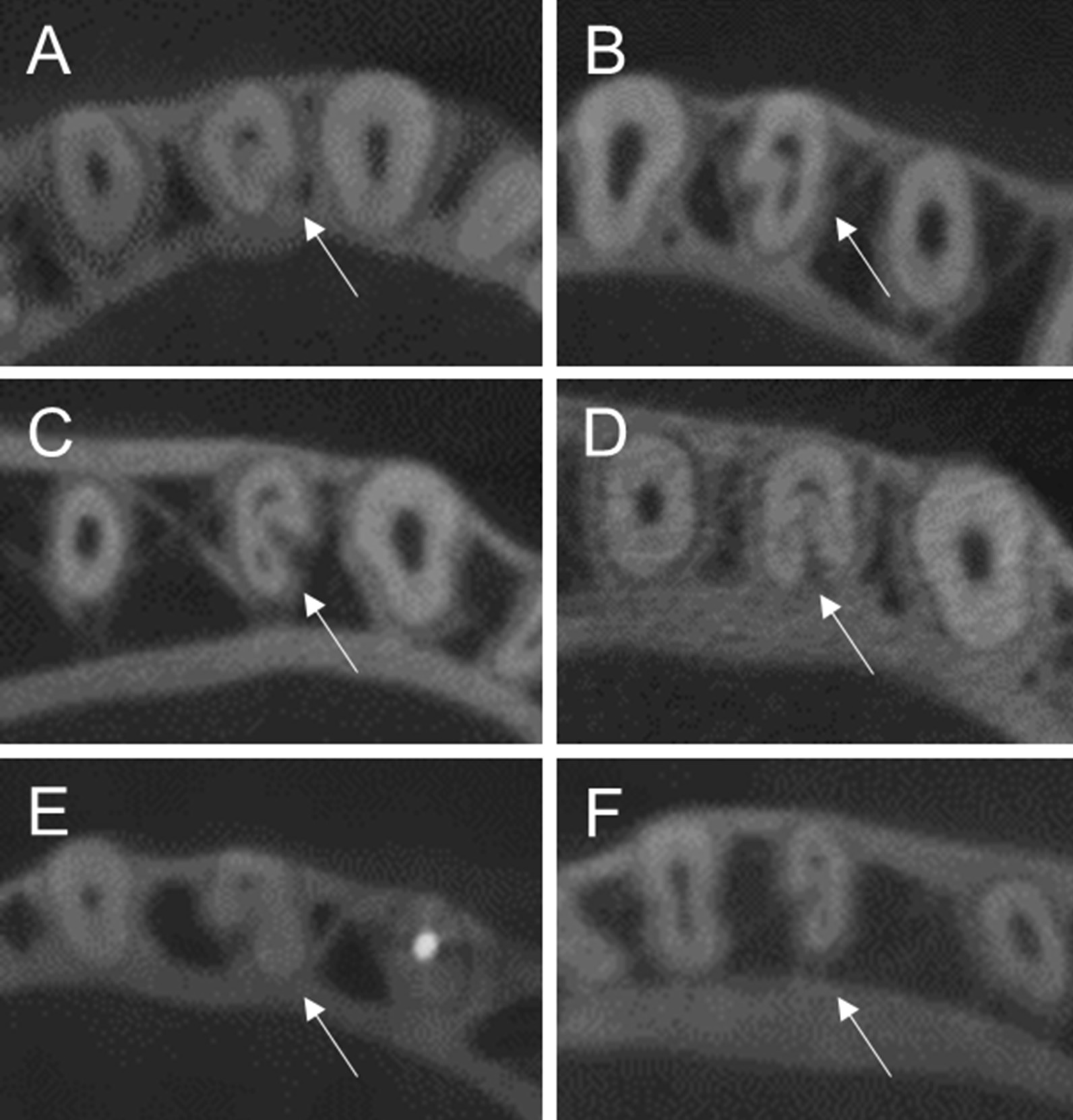
Axial views of CBCT images demonstrate C-shaped root canals in mandibular premolars **A**, **B** Category I, **C**, **D** Category II, **E**, **F** Category III. (Arrows indicate C-shaped root canals in mandibular premolars)

This study used the cementoenamel junction (CEJ) level as an initial reference point on the root canal and recorded the level of root canal branching by dividing the root canal into 3 levels: coronal, middle, and apical. Moreover, the criteria for being a C-shaped root canal were having a radicular groove together with at least one cross-section of the root canal belonging to category I (C1): a continuous C with no separation, category II (C2): a semicolon with at least one root canal orifice angle no less than 60 degree, and category III (C3): two or three separated canals with a root canal orifice angle no more than 60 degree [23] (Fig. 2).

The intra-observer reliabilities and inter-observer reliability were assessed using 40 random CBCT images for reevaluation by SK and PT 3 weeks after the first evaluation. The intraclass correlation coefficient was used for intra-observer reliability assessment and Randolph's Free marginal kappa (2005) was used for inter-observer reliability assessment.

The data were reported using descriptive statistics: the prevalence of root canal configuration according to Vertucci classification, number of roots and their locations in case of more than 1 root, the prevalence of root canal complexities, including a bifurcation, trifurcation or C-shaped root canal, level of branching from the main root canal in the teeth with multiple root canals, appearance of the radicular groove and its location, and bilateral appearance of the same root canal configuration. The relationship between the prevalence of the root canal complexities and sex was analyzed using the chi-squared test.

## Results

The study comprised 1,159 CBCT images of 349 patients (148 males and 201 females) containing 621 mandibular first premolars and 538 mandibular second premolars (Additional file 1). There were 279 CBCT images with bilateral mandibular first premolars, while 218 images had bilateral mandibular second premolars.

The intra-observer reliabilities were found to be good to almost perfect with an intraclass correlation coefficient value 0.80–1 and 0.89–1 for PT and SK, respectively. The inter-observer reliability was found to be good to almost perfect with Randolph's Free marginal kappa (2005) value 0.72–0.91.

When evaluating the number of roots, the mandibular first and second premolars typically had 1 root (98.1% and 99.8%, respectively), while 2 roots were found in 1.6% and 0.2% of the mandibular first and second premolars, respectively. In addition, 3 roots were found in only 0.3% of the mandibular first premolars (Table 1). The mandibular premolars with two roots had mesial and distal roots and buccal and lingual roots at 63.6% and 36.4% respectively, while three roots were composed of two buccal roots and one lingual root.

**Table 1:** The number of roots in mandibular first and second premolars

Tooth	Sample size (n)	Number of roots		
		1 root n (%)	2 roots n (%)	3 roots n (%)
Mandibular 1st premolar	621	609 (98.1)	10 (1.6)	2 (0.3)
Mandibular 2nd premolar	538	537 (99.8)	1 (0.2)	–
Total	1159	1146 (98.9)	11 (0.9)	2 (0.2)

The most common root canal configuration was Vertucci type I, with a 63.1% and 98% prevalence in the mandibular first and second premolars, respectively. The second most common root canal configuration was Vertucci type V, or bifurcation, with a prevalence of 28.5% and 1.5% in the mandibular first and second premolars, respectively. The prevalence of other root canal configurations according to Vertucci classification were shown in Table 2.

**Table 2:** Vertucci classification, additional root canal configurations, and C-shaped root canals in mandibular first and second premolars

Vertucci classification	Total		Mand. 1st PM		Mand. 2nd PM	
	Vertucci, n (%)	C-shaped, n (%)	Vertucci, n (%)	C-shaped, n (%)	Vertucci, n (%)	C-shaped, n (%)
Type I (1)	919 (79.3)	–	392 (63.1)	–	527 (98)	–
Type II (2-1)	10 (0.9)	–	9 (1.4)	–	1 (0.2)	–
Type III (1-2-1)	18 (1.6)	7 (0.6)	16 (2.6)	6 (1)	2 (0.4)	1 (0.2)
Type IV (2)	4 (0.3)	–	4 (0.6)	–	–	–
Type V (1-2)	185 (16)	133 (11.5)	177 (28.5)	130 (20.9)	8 (1.5)	3 (0.6)
Type VI (2-1-2)	2 (0.2)	–	2 (0.3)	–	–	–
Type VII (1-2-1-2)	1 (0.1)	–	1 (0.2)	–	–	–
Type VIII (3)	–	–	–	–	–	–
*Additional root canal configurations*						
Trifurcation (1–3)	20 (1.7)	11 (0.9)	20 (3.2)	11 (1.8)	–	–
Total	1159	151 (13)	621	147 (23.7)	538	4 (0.7)

**Fig. 3:**
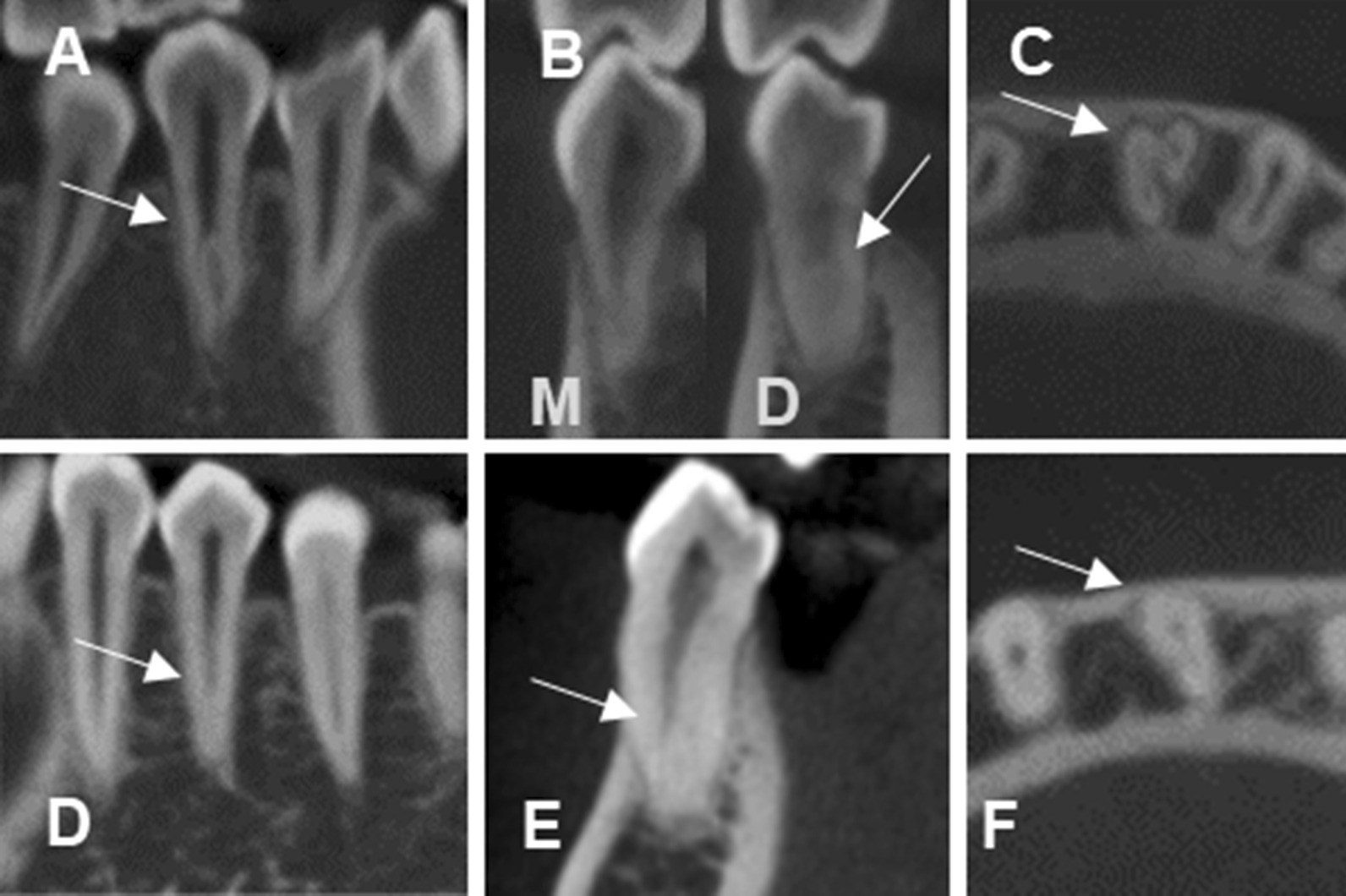
**A**–**C** CBCT images of a mandibular first premolar with three root canals. **A** Sagittal view of a 2-rooted mandibular first premolar with mesial and distal roots. **B** Coronal views of a mesial root with 1 root canal and distal root with 1 root canal extending from the pulp chamber and dividing into 2 root canals at the middle 1/3 level. M indicates mesial root and D indicates distal root. **C** Axial view of mesial, distobuccal, and distolingual root canals. **D**–**F** CBCT images of a mandibular first premolar with two root canals. **D** Sagittal view of a single-rooted mandibular first premolar whose root canal initiates at the CEJ and disappears in the apical third level (fast break). **E** Coronal view of 1 root canal extending from the pulp chamber and dividing into 2 root canals at the middle 1/3 level. **F** Axial view of 2 root canals at the middle 1/3 level. Arrows indicate mandibular first premolars

The prevalence of a bifurcation was 28.5% and 1.5% in the mandibular first and second premolars, respectively. The prevalence of a trifurcation was 3.2% in the mandibular first premolar (Fig. 3), and the prevalence of a C-shaped root canal was 23.7% and 0.7% in the mandibular first and second premolars, respectively (Table 2).

The level of branching was mostly found at the middle 1/3, with 75.5% and 50% in the mandibular first and second premolars, respectively. Coronal branching was found in 15.3%, and 20% of the mandibular first and second premolars, respectively. Teeth with apical branching presented in 9.3% and 30% of the mandibular first and second premolars, respectively (Table 3).

**Table 3:** Level of root canal division in mandibular first and second premolars

Tooth	Level		
	Coronal 1/3 n (%)	Middle 1/3 n (%)	Apical 1/3 n (%)
Mandibular 1st premolar	33 (15.3)	163 (75.5)	20 (9.3)
Mandibular 2nd premolar	2 (20)	5 (50)	3 (30)

A C-shaped root canal was found in two types of root canal configurations according to the Vertucci classification. The 20.9% and 0.6% of C-shaped root canals in the mandibular first and second premolars, respectively, were found to be Vertucci type V. Total of 1% and 0.2% of the C-shaped root canals in the mandibular first and second premolars, respectively, were found to be Vertucci type III. Moreover, 1.8% of the C-shaped root canals in the mandibular first premolar were found to be trifurcations (Table 2).

**Fig. 4:**
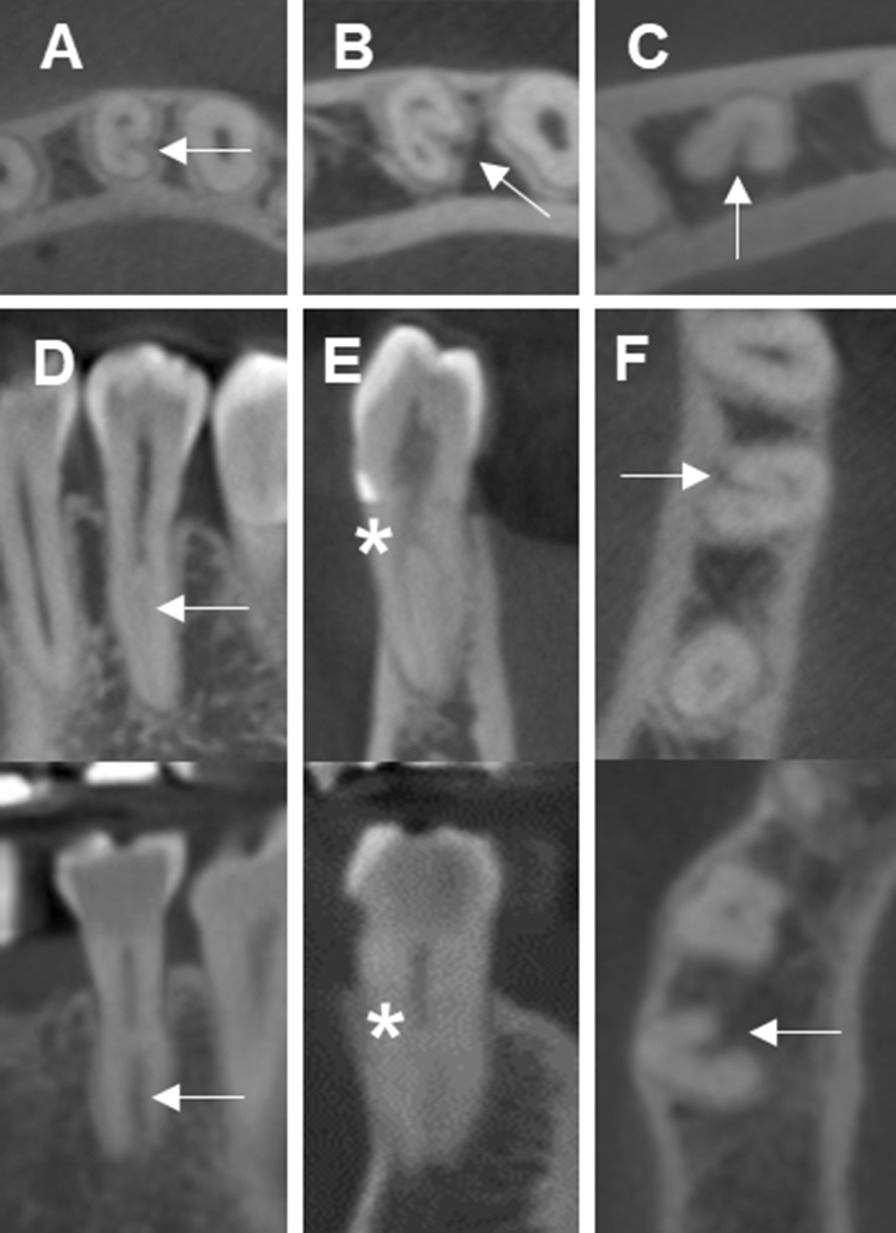
**A**–**C** Axial view of CBCT images show the location of radicular grooves in C-shaped root canals in **A** Mesial, **B** Mesiolingual, and **C** Lingual aspects of the root. **D** Sagittal view of CBCT images show a radiolucent longitudinal line between the mesial and distal part of the root. **E** Coronal view of CBCT images show single-rooted premolars with 1 root canal extending from the pulp chamber and dividing into 2 root canals. **F** Axial view of CBCT images show C-shaped root canals with a lingual radicular groove. (Arrows indicate radicular grooves; asterisks indicate where the root canal divides from 1 into 2 root canals)

Radicular grooves were found in 14.2% and 0.4% of the mandibular first and second premolars, respectively. The radicular groove locations were mostly found at the mesial and mesiolingual aspect of the root, accounting for 55% and 31.8%, respectively. Other locations of the radicular grooves were 10.6% lingual, 0.7% buccal, 0.7% buccal and lingual, and 1.3% mesial and buccal (Fig. 4). In addition to a C-shaped root canal, radicular grooves were also found in other root canal configuration (Table 4).

**Table 4:** The presence of a radicular groove in each Vertucci classification and teeth

Vertucci classification	Radicular groove (%)
Type I	6.4
Type III	4.6
Type IV	1.2
Type V	80.9
Trifurcate (1–3)	6.9

Tooth	Radicular groove (%)
Mandibular 1st premolar	14.2
Mandibular 2nd premolar	0.4

**Fig. 5:**
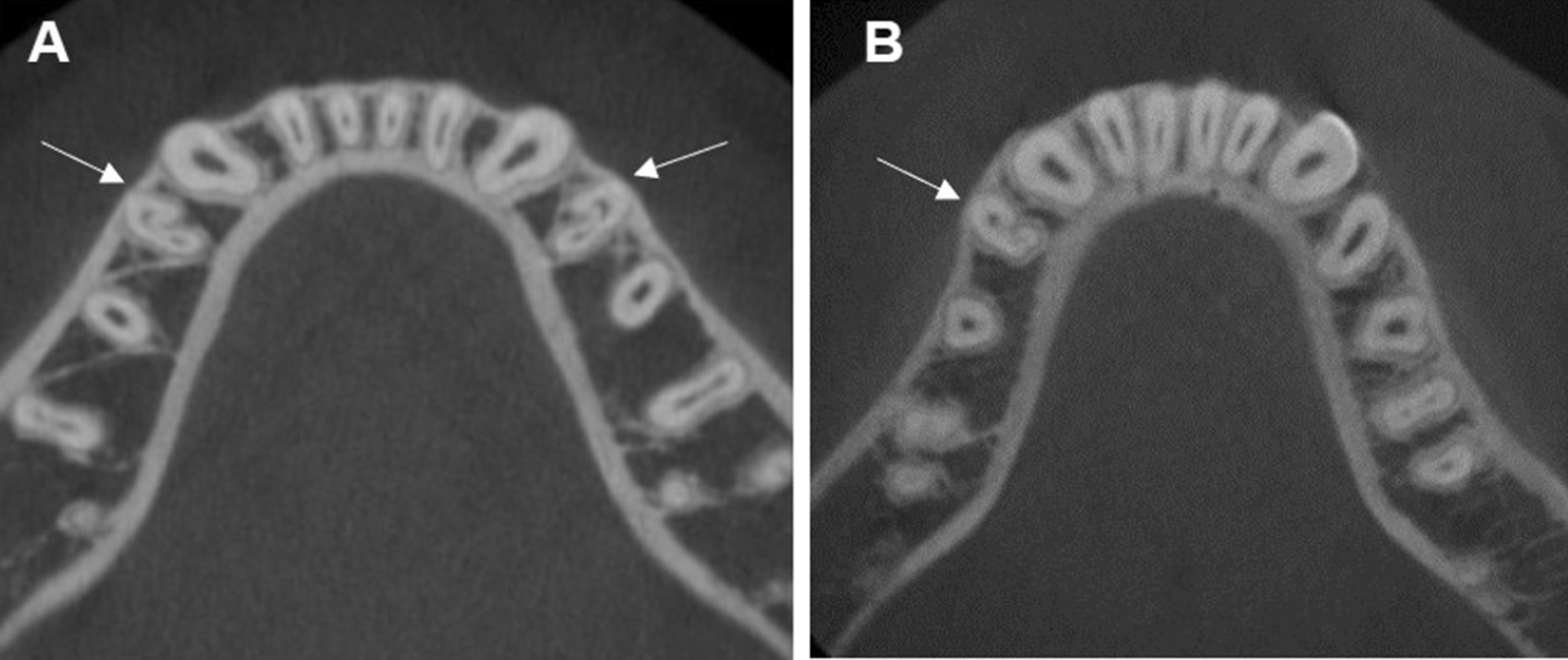
**A** Bilateral appearance of a C-shaped root canal in mandibular first premolars. **B** Unilateral appearance of a C-shaped root canal in mandibular first premolars. (Arrows indicate mandibular first premolars)

Bilateral appearance of the same root canal configuration was identified in 80.3% and 95.9% in the mandibular first and second premolars, respectively. In addition, bilateral appearance of a bifurcation was found in 55.2% of the mandibular first premolar, while bilateral appearance of a bifurcation was not found in the mandibular second premolars. Bilateral appearance of a trifurcation was found in 58.3% of the mandibular first premolars. Moreover, bilateral appearance of a C-shaped root canal was found in 61.4% of the mandibular first premolars, while bilateral appearance of a C-shaped root canal was not found in the mandibular second premolars (Fig. 5).

Of the 349 patients (148 males and 201 females), bifurcations were found in 18.2% and 14.5% of males and females, respectively. Trifurcations were found in 2.4% and 1.2% of males and females, respectively. A C-shaped root canal was found in 14.9% and 11.7% of males and females, respectively. The chi-squared test results indicated that there was no relationship between sex and the prevalence of a bifurcation, trifurcation, or C-shaped root canal with p-values of 0.093, 0.364, and 0.106, respectively.

## Discussion

The present study evaluated the prevalence of a range of mandibular premolar root and root canal configurations in an adult Thai population. We found that the most common root canal configuration was Vertucci type I, with a 63.1% and 98% prevalence in the mandibular first and second premolars, respectively. Although most mandibular first and second premolars were classified as Vertucci type I, we found that the mandibular first premolars were also classified as Vertucci type V (28.5%). This is consistent with previous studies that found 21.91–24% Vertucci type V mandibular first premolars [11–13, 16, 17, 24]. A previous study in a Thai population found 16.9% Vertucci type V mandibular first premolars [21]. These findings emphasize the importance of searching for the second root canal in mandibular first premolars to obtain complete debridement and obturation of the root canal system.

More than 98% of the mandibular premolars had a single root. This was consistent with previous studies where a single root was found in more than 97% of mandibular premolars [5, 6], and a previous study in a Thai population found that 94.27% and 100% of mandibular first and second premolars, respectively had a single root [21]. When there are multiple root canals in a single root, root canals can be missed due to the difficulty in identifying the extra canal on the periapical radiograph. In 2018, the American Association of Endodontists recommended using CBCT imaging for the initial treatment of teeth with the potential for extra canals and suspected complex root canal morphology, including mandibular premolars [25]. However, the ALARA (As Low As Reasonably Achievable) principle should be followed. CBCT imaging should be used only when a two-dimensional periapical radiograph cannot provide sufficient information for adequate root canal treatment. Moreover, a limited field of view CBCT that is slightly larger than the area of interest should be used to reduce the radiation dose and obtain a higher spatial resolution CBCT image [26]. A CBCT image provides important information about tooth morphology, number and location of the root canals, details of the root canal, such as calcification, curvature, and other complex root canal morphology, and external root morphology, such as a root concavity. This information can warn the dentist that these teeth will be challenging to debride and obturate.

We found that mandibular first premolars have more anatomical variations compared with mandibular second premolars in terms of the number of roots and the root canal configuration, similar to previous studies [5, 6, 21]. The dentist should be aware of the anatomical variations in mandibular premolars, especially the mandibular first premolar. In addition to CBCT imaging, there are multiple ways to identify extra canals, including modified horizontal beam angulation of the periapical radiograph, dental operating microscope, Krasner and Rankow laws [4], and knowledge of mandibular premolar root canal configurations.

In a mandibular premolar with more than 1 root, the extra root can be seen on a two-dimensional periapical radiograph. This study found 2 roots in 1.6% and 0.2% of mandibular first and second premolars, respectively. This was consistent with previous studies that found 2 roots in 1.8 and 0.3% of mandibular first and second premolars, respectively [5, 6]. However, a previous study in a Thai population found 2 roots in 4.9% of mandibular first premolars [21]. 63.6% of two-rooted mandibular premolars had mesial and distal roots, which can be seen on a parallel periapical radiograph. Furthermore, 36.4% of two-rooted mandibular premolars had buccal and lingual roots that can be seen using horizontal tube shift x-ray technique or CBCT. There were 3 roots in 0.3% of mandibular first premolars, while no three-rooted mandibular second premolars were found, which was consistent with previous studies that found 3 roots in 0.2% of the mandibular first premolars, and no three-rooted mandibular second premolars were found [5, 6]. A previous study in a Thai population also found 3 roots in 0.9% of mandibular first premolars, and no three-rooted mandibular second premolars [21]. All of the three-rooted mandibular first premolars in this study had mesiobuccal, distobuccal, and lingual root canals that can be seen on a parallel periapical radiographs.

**Fig. 6:**
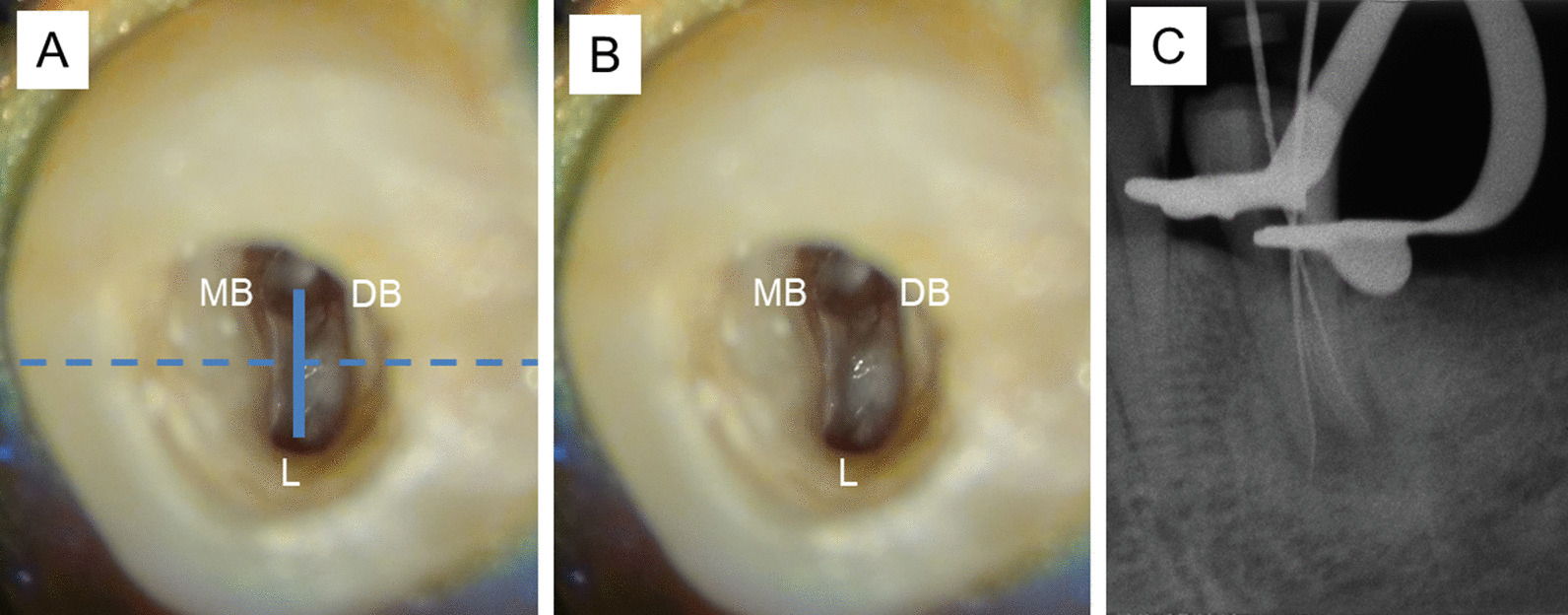
Five Krasner and Rankow laws can be applied in trifurcated left mandibular first premolars, **A** Law of symmetry 1: the mesiobuccal (MB) and distobuccal (DB) root canal orifices are equidistant to the lingual (L) orifice on a line draw in a mesial-distal direction across the center of the floor of the pulp chamber (M-D line), Law of symmetry 2: the orifices of the canals lie on a line perpendicular to M-D line. **B** Law of Color Change: the color of the pulp-chamber floor is always darker than the walls, Law of Orifice Location 1 and 2: the orifices of the root canals are located at the angles in the floor-wall junction, and Law of Orifice Location 3: the orifices of the root canals are located at the terminus of the root developmental fusion lines, **C** a radiograph with files inside the root canals from left to right are mesiobuccal (MB), distobuccal (DB), and lingual (L) root canal respectively. Dashed line indicates M-D line and the solid line indicates the line perpendicular to M-D line

**Fig. 7:**
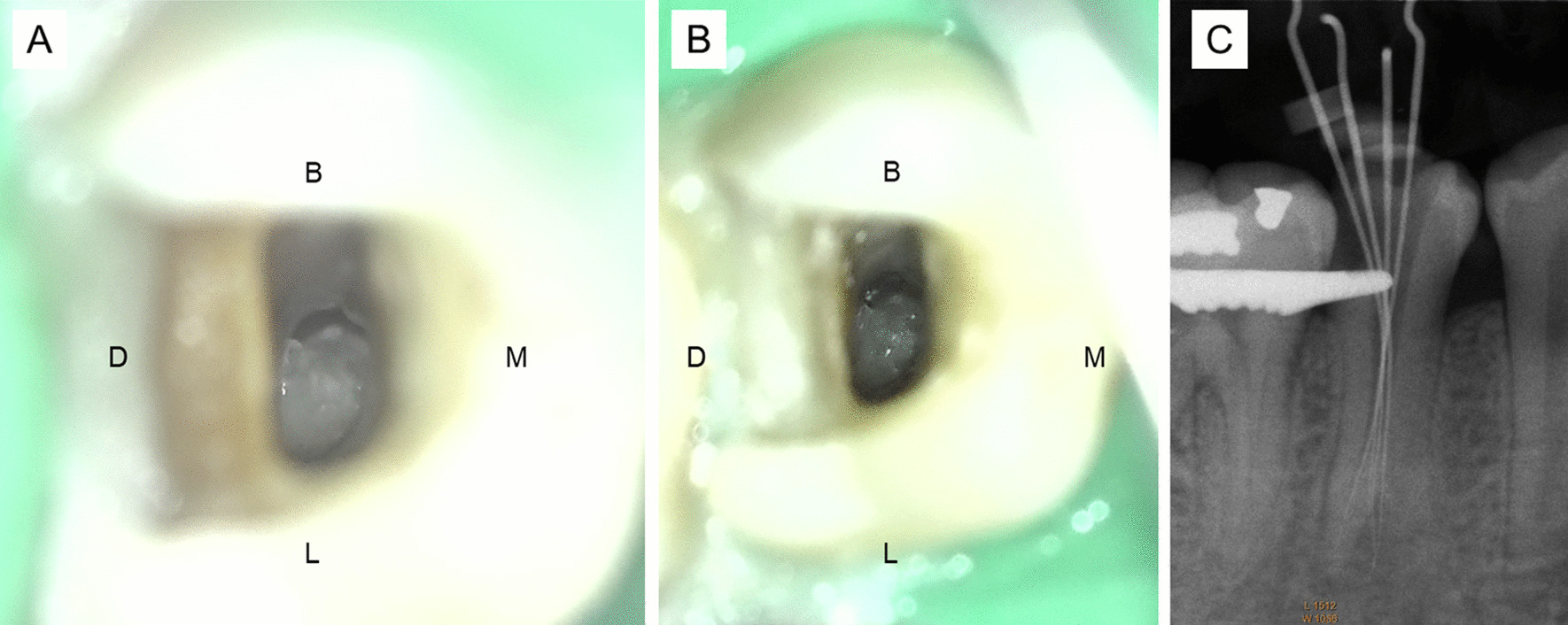
Right mandibular second premolar with a C-shaped canal configuration. **A** A semicolon shape (C2 category), **B** Two Krasner and Rankow laws can be applied in this tooth, Law of Color Change: the color of the pulp-chamber floor is always darker than the walls and Law of Orifice Location 1: the orifices of the root canals are always located at the junction of the walls and the floor **C** a radiograph with files inside the root canals from left to right are one file in the distolingual canal and 3 files in the buccal canal respectively. M, D, B, and L indicate mesial, distal, buccal and lingual aspect of the tooth respectively

Krasner and Rankow laws are used as a clinical guide for pulp chamber and root canal orifice location [4]. However, 3 laws in “Relationships of the pulp chamber to clinical crown” which are applied at the CEJ level cannot be used to identify most of the mandibular premolar extra root canal orifice locations on the pulp chamber floor because this study found that only 1.4% (Vertucci type II, IV, and VI) of the extra root canal orifices initiated at the CEJ level. Moreover, these laws cannot be used to identify a C-shaped canal because this study found that no C-shaped root canal orifice was initiated at the CEJ level (All C-shaped mandibular premolars in this study were Vertucci type III and V). On the other hand, 5 laws in “Relationships on the pulp chamber floor” can be used with a dental operating microscope to identify an extra root canal orifice in a mandibular premolar (Fig. 6). However, only the law of color change and law of orifice location 1 in “Relationships on the pulp chamber floor” can be used with a dental operating microscope to identify a C-shaped root canal orifice (Fig. 7). Because most of the extra root canal orifices in the mandibular premolars in this study initiated apical to the CEJ, illumination and magnification from a dental operating microscope is important for visualizing the deep part of the root canal system.

When extra canals were suspected, such as a bulb-shaped external root anatomy in the periapical radiograph, investigation of extra canals by scouting thoroughly at the internal root canal wall is recommended. Our results indicated that there is a high probability of finding an extra canal branching from the main root canal in the middle third level of the mandibular first and second premolar at 75.5% and 50%, respectively. Extra canal branching was also mostly found in the middle third of the root canal in a previous Thai population study at 76.5% and 80% in mandibular first and second premolars, respectively [21].

C-shaped root canals were also found in mandibular premolars. The results indicated that a C-shaped root canal was present in 23.7% of mandibular first premolars and 0.7% of mandibular second premolars. A previous study in a Thai population found a C-shaped root canal in only 3.7% of mandibular first premolars and 0.5% of mandibular second premolars. The prevalence of multiple root canals and C-shaped root canals in the present study was higher than the previous study in a Thai population [21]. The higher sample number in our study increased the possibility of finding additional root canal variations. Moreover, this study included C3 in the C-shaped root canal criteria, while the other study did not. The present study included C3 as a C-shaped root canal based on previous studies [20, 27–29].

A mandibular premolar with a C-shaped root canal is a mandibular premolar with multiple canals in the C1, C2, and C3 category together with a radicular groove. We found that 64.2% of mandibular first premolars with multiple canals have a C-shaped root canal, while 36.4% of mandibular second premolars with multiple canals had a C-shaped root canal. This prevalence implied that there might be a C-shaped root canal in a mandibular premolar with multiple canals; moreover, a C-shaped root canal can be obscured by a C1, C2, or C3 configuration that may not be observed on the pulp chamber floor of some teeth [30]. Thus, a mandibular premolar with multiple canals should be endodontically treated as a C-shaped root canal to avoid treatment failure due to untreated complex root canal morphology and the isthmus between C-shaped root canals.

Root canal orifice location in a C-shaped mandibular premolar can be done using the same method as for mandibular premolars with multiple canals, using the information from a periapical radiograph and/or CBCT image, root canal scouting thoroughly at the internal root canal wall, and in anticipation based on a contralateral tooth with a known root canal configuration. However, the mechanical instrumentation and root canal obturation of a C-shaped mandibular premolar requires extra care and additional treatment to completely treat a C-shaped root canal without causing iatrogenic complications. Mechanical instrumentation using an anti-curvature filing method is crucial to prevent strip perforation in the radicular groove area.

A C-shaped root canal cross-section has a tapering margin and root canal preparation until the root canal has a round cross-section is at risk of strip perforation. Thus, filing the narrow area and isthmus with a small size file using an antibacterial irrigant and supplemented with irrigation activation using sonic or ultrasonic treatment are important for disinfecting the root canal system without causing damage to the root structure. A thermoplasticized gutta-percha technic was recommended to use in C-shaped root canal obturation with a high flowability root canal sealer and ultrasonic activation of the root canal sealer to fill narrow areas of the root canal and isthmus [31]. Epoxy resin-based root canal sealer AH plus and MTA-based root canal sealer MTA Fillapex were recommended for C-shaped root canal obturation because they have high flowability and were not affected by heat during root canal obturation with a thermoplasticized gutta-percha technic [32–34].

A radicular groove was one of the criteria for root canal classification as a C-shaped root canal. Radicular grooves were most commonly found at the mesial and mesiolingual aspects of the root, similar to previous studies [19, 35, 36]. Although the radicular grooves were most commonly found at the mesial and mesiolingual aspects, mechanical instrumentation using an anticurvature filing method to prevent strip perforation in the radicular groove area should be evaluated on a case-by-case basis [37].

In addition to C-shaped root canals, radicular grooves were most commonly found in bifurcations and in multiple canals, while radicular grooves were found only in 6.4% of single canals (Table 4). These findings are similar to a previous study that reported a significantly higher prevalence of the radicular groove in multiple and complex canals compared with single canals [36]. Having a radicular groove in a tooth with multiple canals and C-shaped root canal can be explained by the role of the epithelial diaphragm of Hertwig’s epithelial root sheath in guiding the shape and number of roots. When the entire circumference of the diaphragm grows evenly and merges, a single root forms with the entire dental papilla inside that will become a single root canal. In contrast, when two areas of the diaphragm opposite one another grow inward rapidly and merge, the dental papilla will be separated into two parts and become 2 root canals, and if multiple areas of the diaphragm grow inward rapidly and merge the dental papilla will be separated into multiple parts and become multiple root canals [38]. However, failure of the inward rapidly growing diaphragms to merge will cause an incompletely-separated dental papilla between diaphragms that will become an isthmus in a C-shaped root canal. In multiple root canals and C-shaped root canal formation, the external surface of the inward rapidly growing diaphragm will become the radicular groove.

This study found 80.3% and 95.9% bilateral appearance of the same root canal configuration of the mandibular first and second premolar, respectively. Corresponding with a previous study, the results showed that bilateral appearance of the same root canal configuration was found at 80.5% in mandibular premolars [39]. These results imply that a contralateral tooth with a known root canal configuration can be used as a clue in anticipating the root canal configuration in the ipsilateral tooth.

This study found a higher prevalence of root canal variations in male patients. A previous study in a Thai population also found that males had a significantly higher prevalence of root canal variations compared with females [21]. However, there were no significant differences in root canal variations between male and female patients in the present study, thus, sex cannot be used to determine whether the tooth has root canal variations.

The limitation of the present study is that CBCT images with large field of view at 100 × 100 mm and 100 × 50 mm with 0.25 mm voxel size were included for evaluation to investigate the bilateral appearance of the same root canal configuration. CBCT images with a large field of view have less resolution compared with a smaller field of view. However, the 0.25 mm voxel size CBCT image is sufficient for evaluating root canal configurations. A previous study found that the second mesiobuccal canal (MB2 canal) detection with CBCT images of 0.3, 0.25, and 0.2 mm voxel size were similar, in the absence of a root canal filling material in the first mesiobuccal canal (MB1 canal) [24].

## Conclusions

In conclusion, dentist should be aware of the high anatomical variability and complexity of mandibular premolars, especially mandibular first premolars. Horizontal tube shift x-ray technique, CBCT, dental operating microscope, and knowledge of root canal configurations have an important role in root canal identification in a mandibular premolar with suspected complex root canal morphology. Use of these will reduce the likelihood of an untreated root canal that leads to endodontic failure. Moreover, a mandibular premolar with multiple root canals should be treated as if there was a C-shaped root canal to completely treat the tooth without causing iatrogenic complications. When a root canal variation was found on one side, it is possible that the contralateral tooth will have a similar root canal variation, especially in mandibular first premolars.

## Supplementary Information

**Additional file 1.** Raw data of CBCT images.

## Data Availability

The datasets generated and analyzed during the current study are available in supplementary file. Other images are available from the corresponding author on reasonable request.

## Abbreviations

TN: Tooth number
CBCT image: Cone-beam computed tomographic image
CEJ: Cementoenamel junction
MB: Mesiobuccal
DB: Distobuccal
L: Lingual
M-D line: A line draw in a mesial-distal direction across the center of the floor of the pulp chamber
MB2 canal: The second mesiobuccal canal
MB1 canal: The first mesiobuccal canal
